# Geographical mapping of a multifocal thyroid tumour using genetic alteration analysis & miRNA profiling

**DOI:** 10.1186/1476-4598-7-89

**Published:** 2008-12-04

**Authors:** Sinéad T Aherne, Paul C Smyth, Richard J Flavin, Susan M Russell, Karen M Denning, Jing Huan Li, Simone M Guenther, John J O'Leary, Orla M Sheils

**Affiliations:** 1 Department of Histopathology, Trinity College, Dublin, Ireland; 2 Applied Biosystems, Foster City, California, USA; 3 Central Pathology Laboratory, St James's Hospital, Dublin, Ireland

## Abstract

**Background:**

Papillary thyroid carcinoma (PTC) frequently presents as multiple tumour-foci within a single thyroid gland or pluriform, with synchronous tumours comprising different histological variants, raising questions regarding its clonality. Among the genetic aberrations described in PTC, the *BRAF *V600E mutation and *ret/PTC *activation occur most commonly. Several studies have investigated the genetic alteration status of multifocal thyroid tumours, with discordant results.

To address the question of clonality this study examined disparate geographical and morphological areas from a single PTC (classic PTC, insular and anaplastic foci, and tumour cells adjacent to vascular invasion and lymphocytic infiltrate) for the presence of *ret/PTC 1 *or *BRAF *mutations. Moreover, we wanted to investigate the consistency of miRNA signatures within disparate areas of a tumour, and geographical data was further correlated with expression profiles of 330 different miRNAs. Putative miRNA gene targets were predicted for differentially regulated miRNAs and immunohistochemistry was performed on tissue sections in an effort to investigate phenotypic variations in microvascular density (MVD), and cytokeratin and p53 protein expression levels.

**Results:**

All of the morphological areas proved negative for *ret/PTC 1 *rearrangement. Two distinct foci with classic morphology harboured the *BRAF *mutation. All other regions, including the insular and anaplastic areas were negative for the mutation.

MiRNA profiles were found to distinguish tumours containing the *BRAF *mutation from the other tumour types, and to differentiate between the more aggressive insular & anaplastic tumours, and the classic variant. Our data corroborated miRNAs previously discovered in this carcinoma, and additional miRNAs linked to various processes involved in tumour growth and proliferation.

**Conclusion:**

The initial genetic alteration analysis indicated that pluriform PTC did not necessarily evolve from classic PTC progenitor foci. Analysis of miRNA profiles however provided an interesting variation on the clonality question. While hierarchical clustering analysis of miRNA expression supported the hypothesis that discrete areas did not evolve from clonal expansion of tumour cells, it did not exclude the possibility of independent mutational events suggesting both phenomena might occur simultaneously within a tumour to enhance cancer progression in geographical micro-environments within a tumour.

## Background

Thyroid cancer is the most common endocrine malignancy and accounts for the majority of endocrine cancer deaths each year [1]. Carcinomas of the thyroid comprise a heterogeneous group of neoplasms with distinctive clinical and pathological characteristics. Most thyroid cancers originate from the follicular cell epithelium. The majority of tumours are designated as papillary thyroid cancer (PTC) and are well differentiated, indolent and are associated with good prospects of survival. Undifferentiated or anaplastic carcinomas, however, are very aggressive with survival rates of less than one year in most instances [2].

Thyroid cancer harbours several highly prevalent genetic alterations including *ret/PTC *rearrangements, *Ras *mutations, and *BRAF *V600E point mutations [3]. There have been intriguing associations between the morphology of PTC and specific molecular findings. In the past our group and others have noted an association between classic PTC morphology and the *BRAF *V600E mutation and between variant morphology and *ret *rearrangements particularly *ret/PTC 3 *[4,5]. Similarly *ret/PTC 3 *appears to strongly correlate with the solid/follicular variant seen commonly in children exposed to Chernobyl fallout [6].

PTC frequently presents as multiple tumour-foci within a single thyroid gland. In addition, a significant proportion of PTC are also pluriform, with synchronous tumours comprising of different histological variants. These PTC foci may either arise from intrathyroidal metastasis of a clonal population of cells or as a consequence of multiple tumours arising independently. Several studies have been performed using various techniques in an effort to elucidate whether most poorly differentiated anaplastic carcinomas develop through a multi step carcinogenesis process from the well differentiated thyroid cancer subtypes, or if they arise independently [7-15]. Among genetic aberrations described in PTC, the *BRAF *V600E mutation and *ret/PTC *activation occur most commonly. Most of the studies thus far have focused on investigating the genetic alterations present in the different foci in an effort to determine their origin and potential clonality. Unfortunately these studies have produced conflicting results to date and no consensus has been reached. In order to further advance this research, examination of the regulatory environment of the thyroid along with genetic alteration analysis may prove beneficial.

MicroRNAs (miRNA) are a recently discovered family of short non-protein-coding RNAs with diverse functions, including regulation of cellular differentiation, proliferation and apoptosis [16]. These small non-coding RNAs constitute a novel class of gene regulators that function by negatively regulating gene expression by targeting mRNAs for cleavage or translational repression. To date, 695 human miRNAs have been identified [17], however, evidence suggests the total number of human miRNAs may be much larger. Studies have indicated that different populations of miRNAs are expressed in cancer and normal tissues with general down regulation of miRNAs in tumours, suggesting that tumours may each have a discrete "miRNA signature" [18]. MiRNAs are also thought to be capable of acting as both tumour suppressors and oncogenes. This differential expression of miRNAs between malignant and normal tissues and between different types of tumour may indicate that miRNAs are determinants of clinical diagnostic and prognostic significance. Recent studies in miRNA expression in PTC have found an aberrant miRNA expression profile in PTCs compared with normal thyroid tissues [19-21].

The origin of multifocal thyroid lesions is potentially an important issue in patient treatment. In fact, multicentric cancer has been associated with local and distant metastases, mortality, and tumour recurrence [22]. If multiple foci are found to be clonal in origin due to the intraglandular metastases of a single tumour, it may indicate an increase in the aggressiveness of the cancer & therefore it may be more likely to further metastasize into other tissue. If this is discovered a more aggressive treatment approach could be required.

To expand on the question of clonality, the objective of this study was to examine disparate geographical and morphological areas from a single PTC for the presence of *ret/PTC 1 *or *BRAF *mutations and correlate it with miRNA expression profiles. A multicentric case containing classic PTC, insular and anaplastic foci, and areas of lymphocytic infiltrate and vascular invasion was selected for analysis. The expression of 330 miRNAs was examined using a recently developed technique, which uses stem loop primers for reverse transcription followed by real time TaqMan^® ^PCR [23].

Hierarchical clustering analysis of the profiles produced was carried out, along with miRNA gene target prediction. Immunohistochemistry was also performed on tissue sections in an effort to investigate phenotypic variations in microvascular density (MVD), and cytokeratin and p53 protein expression levels. The varying miRNA profiles within different foci of a single tumour illustrate how a tumour's microenvironment can affect regulatory processes underpinning carcinogenesis, and highlight a caveat that ought to be observed in the search for the discovery of future cancer biomarker signatures, particularly in the context of multifocal tumours. Many miRNA studies to date have highlighted characteristic 'signatures' that associate with particular tumours/phenotypes. In this study we demonstrate that regulatory processes engendered by non-coding RNAs are more subtle and signatures can vary within individual areas of a single tumour. Laser Microdissection ensured that the cells interrogated were homogenous and follicular epithelial in origin, nonetheless their miRNA profiles varied depending on the specific micro-environment from which they were harvested.

## Methods

### Tissue sample

A single archival case of formalin fixed, paraffin-embedded (FFPE) multifocal thyroid tumour was chosen for this analysis. Eight areas were chosen for examination: 1 normal thyroid, 2 classic PTC, 1 insular, 2 anaplastic thyroid carcinomas, 1 area surrounding lymphocytic infiltrate, and 1 surrounding vascular invasion. Haematoxylin and eosin (H&E) sections were reviewed by a histopathologist (RF) and classified according to a recognized system [24]. Corresponding paraffin blocks were then collected from the archives of St. James Hospital. Ethical approval for this study was granted by the SJH/AMNCH Research Ethics Committee.

### Laser capture microdissection

6 *μ*m sections were cut using a microtome (Microm HM 325, Medical Supply Co. Ltd, Ireland) from each paraffin block, mounted on uncharged slides, dewaxed and H&E stained (Tissue-Tek DRS 2000 Autostainer, Sakura, CA, USA). Pure populations of thyrocytes were obtained from each section by laser capture microdissection using the PixCell II System (Acturus Engineering Inc., CA, USA). Laser capture microdissection produces low yet accurate yields of RNA & DNA (yields in the range of 1.7–9 ng/*μ*l of RNA & 3.2–8.8 ng/*μ*l of DNA were achieved in this study). Cells were captured according to a standard protocol: laser spot size = 15 *μ*m, pulse power = 25 mW, pulse width = 1.5 ms, threshold voltage = 285 MV. The total number of pulses in each case was approximately 700 (range = 500–1200). After microdissection the Capsures^® ^(Capsuret Macro LCM caps, LCM 0201, Techno-Path Ltd, Ireland) containing the homogenous cell populations were placed in a sterile microcentrifuge tube ready for RNA & DNA extraction.

### Nucleic acid extraction

DNA & RNA were extracted using the RecoverAll™ Total Nucleic Acid Isolation, Optimized for FFPE Samples Kit (Ambion, Texas, USA), according to the manufacturers' instructions, with the exception of the tissue deparaffinization step of the protocol as the sections had been deparaffinised during the H&E staining. Purified RNA & DNA were eluted in 60 *μ*l volumes and the nanogram concentration per microlitre was verified using a Nanodrop spectrophotometer (ND-1000, Labtech International, UK).

### TaqMan^® ^expression analysis

#### BRAF V600E mutation detection

TaqMan^® ^SNP detection was used for *BRAF *V600E mutation detection as previously described [4]. Primers and probes used in this experiment were designed and used to the manufacturer's recommendations. The primers/probes used were as follows: 5' CAT GAA GAC CTC ACA GTA AAA ATA GGT GAT 3' [BRAF-F], 5' GGA TCC AGA CAA CTG TTC AAA CTG A 3' [BRAF-R], VIC-5' CCA TCG AGA TTT CAC TGT AG 3' [BRAF-PWT], and FAM-5' CCA TCG AGA TTT CTC TGT AG 3' [BRAF-PMUT]. Amplification and analysis was performed using an ABI Prism 7900 HT Sequence Detection System (Applied Biosystems, CA, USA) for 40 cycles (92°C for 15 sec, 60°C for 1 min).

#### Ret/PTC1 rearrangement detection

TaqMan^® ^RT-PCR was used for *ret/PTC 1 *rearrangement detection. Primers and probes used in this experiment were designed and used according to the Applied Biosystems (Foster City, CA, USA) Assays-by-Design SM service. Amplification and analysis was performed on an ABI Prism 7900 HT Sequence Detection System (Applied Biosystems, CA, USA) (48°C for 30 min then 92°C for 15 sec, 60°C for 1 min × 40 cycles).

#### MiRNA analysis

Multiplexing stem-loop RT-PCR with mir-16 as an endogenous control was used to analyse miRNA profiles as previously described [25]. Briefly, the process involves three steps. Steps 1 and 2 are multiplexed reactions with 330 sets of primers for 330 miRNAs. Step 1 reverse transcribes all the miRNAs in a single reaction and then step 2 PCR amplifies the cDNA products to provide enough sample for step 3. Step 3 is done as simultaneous, individual singleplex TaqMan^® ^reactions in 96-well reaction plates to monitor the abundance of each miRNA after the multiplexed RT-PCRs. Sequences for primers and probes will be provided by the authors upon request. As previously, amplification was performed using an ABI Prism 7900 HT Sequence Detection System (Applied Biosystems, CA, USA).

### Data analysis

ΔΔC_T _studies were performed on the miRNA data using the SDS 2.1 software (Applied Biosystems, CA, USA). The studies were then exported to the Microsoft^® ^Office Excel^® ^2003 software (Microsoft Corporation, WA, USA) for further analysis. The primary means of identifying differentially expressed miRNAs was based on fold change. MiRNAs were considered differentially expressed between tumour areas if they possessed fold changes >2 or <0.5. Hierarchical clustering analysis was performed using the Spotfire programme. Clustering analysis was performed using WPGMA and similarity between samples was assessed by correlation analysis. Up & down regulated miRNAs were entered into the three-way intersection in the miRGen database to search for putative miRNA target genes [26]: ). Gene ontology analysis of the putative target gene lists was then performed using the PANTHER database . This enabled the identification of significantly over and under represented pathways in the gene lists.

### Immunohistochemistry

6 *μ*m sections were cut from the archived blocks, deparaffinised, and dehydrated. Sections underwent heat induced epitope retrieval using Trilogy (CellMarque, Greenwood, AR). Immunohistochemical staining, using Monoclonal Mouse Anti-Human cytokeratin (DakoCytomation, Copenhagen, Denmark), CD34 (Serotec, Oxford, UK), and p53 (Novocastra, Newcastle upon Tyne, UK), was performed on the Nexes immunostainer using a strepavidin peroxidise procedure. Antigen-bound primary antibody was detected using strepavidin-biotin immunoperoxidase complex (Ventana *i*-view kit, Ventana Medical Systems). Slides were examined blind by a histopathologist (RF) and classified according to a recognized system [27].

## Results

### Genetic alteration status

TaqMan^® ^RT-PCR was used to detect the presence of *ret/PTC 1 *rearrangement in follicular epithelial cells dissected from each of the eight designated areas (1 normal thyroid, 2 classic PTC, 1 insular, 2 anaplastic thyroid carcinomas, 1 area surrounding lymphocytic infiltrate, and 1 surrounding vascular invasion). RNA from the TPC1 cell line was used as a positive control. All areas proved negative for *ret/PTC 1 *rearrangement.

**Figure 1:**
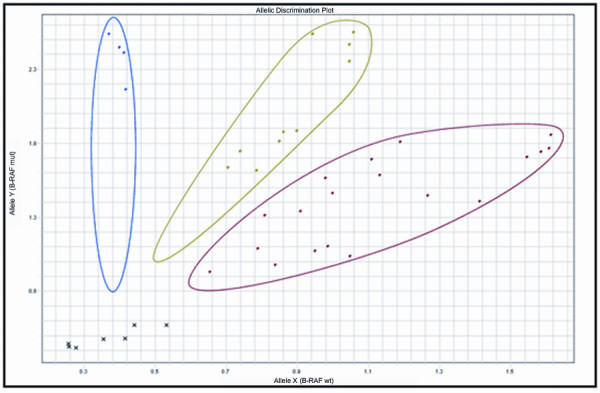
**TaqMan^® ^SNP assays for *BRAF *V600E mutation detection**. DNA from the TPC1, BC-PAP, and K-2 cell lines was used for positive control purposes. TPC1 contains the wild type *BRAF *allele, K-2 is heterozygous for the mutant allele, and BC-PAP is homozygous for the mutant allele. The figure shows clustering of the samples into 3 distinct groups depending on their respective levels of VIC/FAM fluorescence: homozygous T1799A mutation (●), homozygous wild-type/normal (●) and heterozygous T1799 mutation (●). Negative controls and undetermined samples are also displayed (×). The two classic PTC tumour areas are the only ones that exhibit the *BRAF *mutation.

TaqMan^® ^SNP detection was used to detect the presence of the *BRAF *mutation. This assay acts as its own endogenous control as the wild type allele will be detected in the absence of the mutant. The two foci with classic morphology harboured the *BRAF *mutation. All other tumour areas, including the insular and anaplastic areas were negative for the mutation (Figure 1). This result indicates that the anaplastic lesions may not have evolved from progenitor classic PTC foci.

### Regulatory environment analysis

#### Geographical patterns in miRNA expression

**Figure 2:**
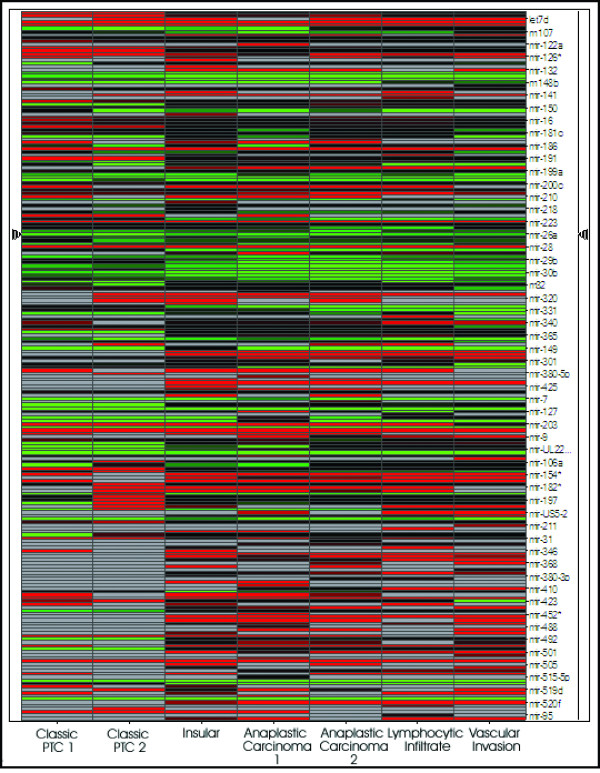
**Heat map of miRNA expression**. Each miRNA listed was considered differentially expressed between tumour areas if they possessed fold changes >2 or <0.5.

Expression levels of 330 miRNAs were analysed in the eight thyroid geographical regions (Figure 2). The ΔΔC_T _method [28] was used to calculate the fold change of miRNA expression between tumour areas, and miRNAs were considered differentially expressed if they possessed fold changes >2 or <0.5. Cells from the area of normal thyroid epithelium were utilised to calibrate the study, and mir-16 was used as an endogenous control.

Figure 2 illustrates the miRNA expression profiles in the different tumour areas. It may be noted from this figure that expression of miRNAs across the tumour areas appears to be non-homogenous. Of the 330 miRNAs, 225 (68%) were expressed in the tissues studied. All of the cancer areas (classic PTC, insular, and anaplastic cancer) showed a general down-regulation in miRNA expression compared to normal thyroid tissue (avg 53.9%) which is the general trend observed when comparing miRNAs in cancer tissues to normal tissue [18]. Interestingly, the cells surrounding the areas of lymphocytic infiltrate and vascular invasion showed a marginal increase in miRNA expression compared to normal thyroid epithelium (avg 53.4%). These cells were captured from an area of anaplastic carcinoma. Perhaps the up-regulation of miRNAs surrounding these areas may enable the down-regulation of gene expression to facilitate angiogenesis and metastasis in this aggressive cancer and assist in its evasion of the immune system (See Additional file 1 for miRNA RQ values).

Table 1 shows the top five up and down regulated miRNAs in each tumour area compared to normal thyroid tissue. This table, in contrast with figure 2, emphasizes the similarities that can be noted between the profiles with mir-221 appearing up-regulated in all tumour areas, and mir-124a and mir-328 appearing prevalently down-regulated in tumour areas. An inevitable element of similarity must be expected in the profiles of these tumour areas as they originate from a single thyroid specimen.

**Table 1:** Five most up and down regulated miRNAs in each tumour area compared to normal thyroid epithelium.

**Up-regulated miRNAs**				
**Classic PTC**	**Insular Carcinoma**	**Anaplastic Carcinoma**	**Lymphocytic Infiltrate**	**Vascular Invasion**

mir-221	mir-221	mir-221	mir-221	mir-221
mir-324-5p	mir-486	mir-205	mir-205	let7e
mir-93	mir-205	mir-324-5p	mir-378	mir-412
mir-182*	let7e	mir-486	let7e	mir-15a
mir-378	mir-320	mir-93	mir-15a	mir-497

**Down-regulated miRNAs**				

**Classic PTC**	**Insular Carcinoma**	**Anaplastic Carcinoma**	**Lymphocytic Infiltrate**	**Vascular Invasion**

mir-124a	mir-124a	mir-328	mir-328	mir-328
mir-192	mir-520e	mir-149	mir-124a	mir-423
mir-UL148D-1	mir-345	mir-520e	mir-192	mir-518a-2*
mir-let-7g	mir-7	mir-375	mir-152	mir-124a
mir-let7f	mir-140	mir-124a	mir-127	mir-152

**Figure 3:**
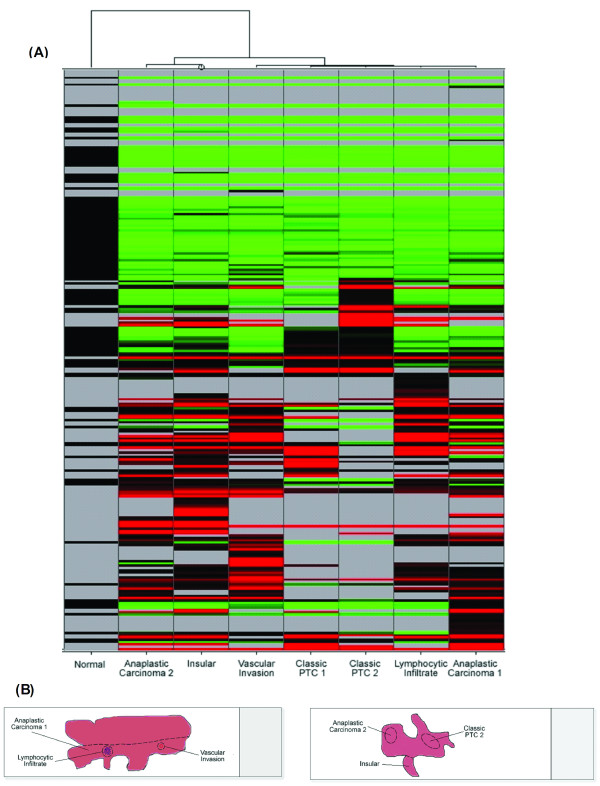
**Hierarchical Clustering of miRNA profiles**. **(A) **Hierarchical clustering diagram of the miRNA profiles of the thyroid tumour areas. **(B) **Images illustrating the geographical location of the tumour foci in respect to each other.

Hierarchical clustering analysis was then performed on the miRNA data to further investigate the potential similarities and differences between the miRNA profiles (Figure 3). The analysis revealed two main clusters, with one cluster dividing into two sub-groups. The normal thyroid epithelium forms one of the main clusters and groups separately to all of the cancer areas. The insular cancer and anaplastic carcinoma 2 areas form one sub-group, and the other tumour areas form the other sub-group. As illustrated from figure 3b, the insular and anaplastic carcinoma 2 samples were excised from regions of close proximity, and the lymphocytic infiltrate, vascular invasion, and anaplastic carcinoma 1 areas were harvested from the same geographical region. This clustering of miRNA profiles corresponding to the geographical position of the cells suggests that the location of the cancer foci within the tumour may influence the miRNA profiles. However as mentioned previously, an element of similarity in profiles must be anticipated in cells adjacent to each other.

In juxtaposition to the geographical clustering of cellular areas, the classic PTC 2 miRNAs sub-group with the other PTC area, separating it from the other foci from the same tissue section (insular and anaplastic carcinoma 2). This, along with possession of the *BRAF *mutation, points towards the similarity of the classic PTC genetic profiles. However the two anaplastic areas divide into separate sub-groups. This raises the question as to why the cancer foci are behaving differently and not grouping together. The grouping of the two classic PTC foci which both contain the *BRAF *mutation indicates that their genetic profiles are similar and may suggest that these two foci may be clonal in origin and have originated from the same mutational event or cascade. However, the anaplastic carcinomas separate into the two sub-groups within the cancer cluster perhaps indicating that these cancers may have arisen independently. Therefore it may not be a question as to whether multifocal tumours arise due to the clonal metastasis of tumour cells or of independent mutational events, but perhaps both phenomena can occur simultaneously within the one tumour to enhance cancer progression.

#### Expression of miRNAs in thyroid tumours containing BRAF mutation

Along with looking at the overall geographical trends of miRNA expression in a multifocal tumour, this data enabled us to view the tumour categorically and explore specific aims and questions. Therefore we consequently looked at distinguishing the *BRAF *containing PTC foci from the other cancer areas. A number of miRNAs were found to be both up and down regulated in the PTC areas in comparison to all the others (Table 2). These profiles indicate that PTC can be further distinguished from the other tumour areas. Furthermore, several of these miRNAs correspond with miRNAs published in thyroid cancer-related papers, and papers relating miRNAs to the tumourigenesis process from our group and others. For instance mir-222 [19,20], mir-106a [29,30], mir-26a [19,29], and mir-34c [31-33].

**Table 2:** Differentially expressed miRNAs in classic PTC tumour areas v all others.

**Up-regulated miRNAs**	**Down-regulated miRNAs**
mir-191*	mir-106b
mir-146b	mir-451
mir-US33-1	mir-26a
mir-151	mir-199a*
mir-193b	mir-193a
mir-222	mir-34c
mir-518a-2*	mir-UL22A-1
mir-106a	mir-492
mir-296	mir-148b
mir-197	mir-UL112-1
mir-328	mir-500
mir-331	mir-UL148D-1
mir-423	mir-let7f
	mir-146a

Other areas include insular and anaplastic, and thyrocytes surrounding lymphocytic infiltrate and vascular invasion.

#### Expression of miRNAs in aggressive thyroid tumours

It is interesting to further investigate and attempt to distinguish between the anaplastic and insular tumours as they are more aggressive than the usually indolent classic PTC. Several miRNAs were found to be both up and down regulated in the anaplastic areas compared to the insular area. Table 3 shows a selection of the miRNAs discovered. It can be noted that the three miRNAs most up-regulated in anaplastic carcinoma compared to the insular tumour have extremely high RQ values (15668.58, 2326.54, 474.51). One might think that these high values could be explained if those miRNAs; mir-140, mir-345, mir-7 respectively, are not expressed in the calibrator sample, however they are expressed in that sample so these extreme values cannot be attributed to that. The three miRNAs most down-regulated in anaplastic carcinoma also have extreme RQ values. In this study miRNA expression was evaluated through relative quantification; therefore these values may result from low expression levels of those miRNAs in the calibrator sample, or very high expression in the tumour areas.

**Table 3:** Differentially expressed miRNAs in anaplastic tumour areas v insular.

**miRNAs Up-regulated in Anaplastic**	**RQ**	**miRNAs Down-regulated in Anaplastic**	**RQ**
mir-140	15668.58	mir-328	0.000003
mir-345	2326.54	mir-375	0.00001
mir-7	474.51	mir-149	0.00002
Mir-124a	23.18	mir-148b	0.067
mir-221	18.07	mir-150	0.170
mir-192	16.39	mir-27b	0.253
mir-99a	12.66	mir-155	0.302
mir-127	8.80	mir-30a-5p	0.356
Mir-let7a	6.30	mir-125b	0.400
mir-let7f	3.75	mir-30d	0.430
Mir-200a	2.50	mir-15b	0.456

The table also shows the fold change in expression.

Some of these differentially regulated miRNAs in the anaplastic carcinomas have also been noted in papers published by our group and others, for instance mir-221 [19,20], mir-200a [20,31,32], mir-155 [29,30,32,34], and mir-125b [20,29,31].

### MiRNAs; biomarkers or crucial biological mediators?

There are currently two main schools of thought as to the utility of these small RNA gene regulators in the advancement of disease treatment. The first, and most common up to now, being to focus on data such as that above and utilize these RNAs as potential new biomarkers. This has resulted in the publishing of numerous papers proposing potential miRNA cancer signatures with the view to improve diagnosis and early detection [19-21].

The other approach is to view these RNAs as the key to a plethora of knowledge regarding the functioning of cells and the body in both normal and disease states. This involves elucidating the gene targets of the miRNAs and therefore their functions and influence in the cell. MiRNAs are already thought to possess oncogenic and tumour suppressor capabilities, however accurately identifying biologically relevant targets that are regulated by individual miRNAs is currently the predominant challenge in this area of research. This task is further complicated by the fact that each miRNA can potentially regulate many mRNA targets and also each mRNA can be regulated by several miRNAs.

To further explore this aspect of miRNA functionality we attempted to investigate potential targets for the miRNAs listed in tables 2 &3. Several publicly available databases exist for prediction of miRNA target genes. The miRGen database was used in this analysis as it allows the production of lists of gene targets common to three target prediction databases (miRanda, PICTAR, & TARGETSCAN) allowing for increased confidence in the target lists produced. The miRGen putative gene target lists were then entered into the PANTHER database in order to identify significantly over and under represented pathways. The binomial statistic was used to estimate this [35]. One could predict that up-regulated miRNAs would down-regulate their target genes which would in turn down-regulate the pathways over represented in that list. From this one could also infer that down-regulated miRNAs would result in an up-regulation of their target genes and pathways. Pathways such as cytoskeletal regulation by Rho GTPases (p = 0.01) and angiogenesis (p = 0.00006) were significantly over-represented in the putative target lists correlating to miRNAs down-regulated in PTC compared to all other areas, and Wnt (p = 0.00009) and angiogenesis (p = 0.003) pathways were significantly over-represented in the putative target lists correlating to miRNAs down-regulated in anaplastic cancer compared to insular cancer (thus inferring their possible up-regulation at the transcriptome level).

**Figure 4:**
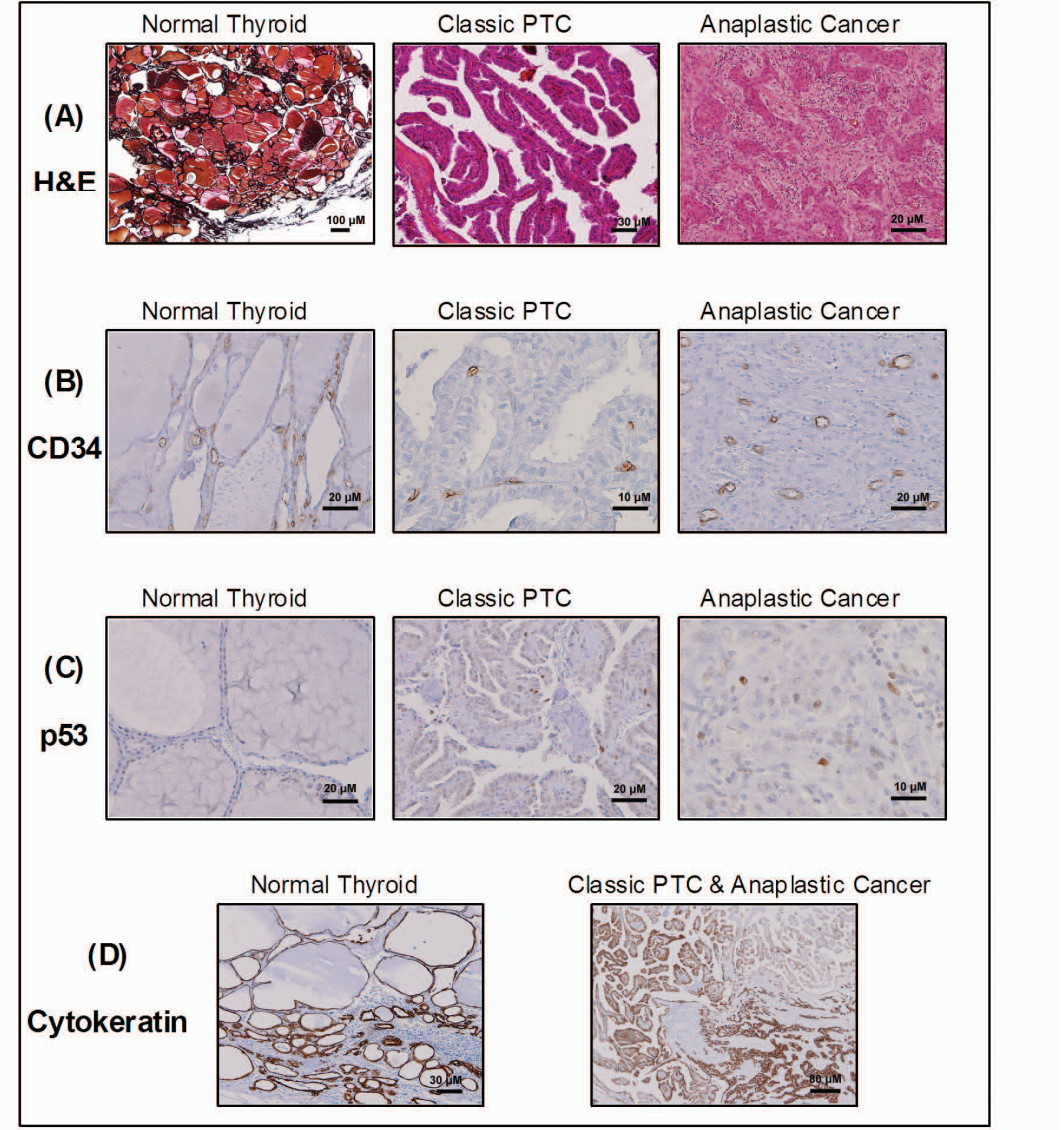
**H&E & immunohistochemistry staining of thyroid sections**. **(A) **H&E stained sections of normal thyroid, classic PTC, anaplastic thyroid cancer, and insular carcinoma. **(B) **Immunohistochemical staining for CD34 in normal, PTC, and anaplastic sections. **(C) **Immunohistochemical staining for p53 in normal, PTC, and anaplastic sections. **(D) **Immunohistochemical staining for cytokeratin in normal thyroid, and in an area showing classic PTC and anaplastic cancer areas.

Although these are predicted results, they may lead us in the right direction in revealing the biological functioning of these miRNAs. Therefore, in an effort to explore the behaviour of some cellular processes across different areas of morphology in a multifocal tumour, we performed some immunohistochemistry on our tissue sections (Figure 4). CD34 was analysed in an effort to assess microvascular density (MVD), cytokeratin to stain epithelial cells and view differentiation status, and p53 as it is commonly mutated in this cancer. Morphological assessment and evaluation of staining intensity may hint at performance of some of the pathways mentioned previously such as angiogenesis and cytoskeletal regulation and wnt signaling.

MVD was marginally increased in anaplastic cancer (n = 197) compared to PTC (n = 131). There are varying reports on the MVD in different thyroid cancers; some show that reduced MVD in poorly differentiated cancer is associated with poor differentiation and worse prognosis, and others demonstrate increased risk of recurrence and shorter disease-free survival with more vascular PTCs [36]. Strong intensity of staining in a high number of cells was observed with the cytokeratin giving an immuno score of 7 for all areas. However there was no staining observed in the squamoid and spindled/sarcomatoid components of the anaplastic cancer illustrating the dedifferentiation of these cells. Finally, there was a marginal increase in p53 between the anaplastic areas (immuno score = 4) compared to the classic PTC areas (immuno score = 3). An activating mutation in the p53 gene could explain this increase in expression as mutated p53 is often found in anaplastic carcinoma [37].

## Discussion

The objective of this study was to examine the question of clonality in multicentric PTC through correlating the miRNA profiles and genetic alteration status of eight tumour areas in a case of multifocal thyroid cancer.

All of the tumour areas examined (one area of normal thyroid, two of classic PTC, one insular, two anaplastic thyroid carcinomas, and thyrocytes surrounding an area of lymphocytic infiltrate and vascular invasion) proved negative for *ret/PTC 1 *rearrangement. The two foci with classic morphology harboured the *BRAF *mutation. All other tumour areas, including the insular and anaplastic were negative for the mutation. This result suggests that the tumours in this thyroid arose independently and not as a result of intrathyroidal metastasis of a clonal population of cells. This outcome also agrees with previous reports supporting this theory. Park [11] and Giannini [8] both studied *BRAF *mutation rate on 61 and 69 patients respectively and their results suggested 39.3% and 40% of the multifocal tumours may have arisen independently. Sugg and colleagues [15] proposed that the majority of the tumours they tested arose independently by showing distinct patterns of *ret/PTC *rearrangement in multifocal PTC. Shattuck and colleagues [13] examined *BRAF *by utilising X-chromosome inactivation on samples from 10 patients and reported discordant genetic patterns in 50% of the multicentric PTC. However, McCarthy [10] also used X-chromosome inactivation and produced results that support the theory of intrathyroidal metastasis. This discordance of conclusions resulting from genetic alteration analysis illustrates that additional analysis would be beneficial in unravelling the tumourigenesis process.

Subsequent to revealing the differential *BRAF *status of the tumour areas, the study of the profiles of 330 miRNAs in the areas provided further insight into multifocal cancer. These miRNA profiles of the different tumour areas highlighted the fact that both similarities and differences could be found between the different foci. The heat-map in figure 2 illustrated the apparent non-homogenous nature of the profiles, however when the most highly up and down regulated miRNAs from each area are viewed (Table 1), the similarities become more evident. This is an encouraging observation as one would expect similarities in the profiles of cells located close to each other, however differences in the profiles of different morphological variants would also be expected, as shown by Nikiforova and colleagues [21]. The down-regulation of miRNAs in the cancer areas compared to normal thyroid epithelium follows trends found generally when comparing cancers to normal tissue [18]. The up-regulation of miRNAs in thyrocytes surrounding areas of lymphocytic infiltrate and vascular invasion is an interesting result as the differential regulation in miRNA and in turn gene expression may aid the progression of the cancer.

The hierarchical clustering analysis performed on the miRNA profiles unearths some exciting findings (Figure 3). The normal and cancerous tissue cluster separately and there is a further sub-grouping within the cancer cluster. Initial examination of the figure may suggest that the tumour areas group together depending on their geographical position within the thyroid. The sites of the different tumour areas captured within the thyroid section can be viewed in figure 3b, and compared to the two sub-groups in which they fall in figure 3a. From this one can see that the anaplastic carcinoma 1, lymphocytic infiltrate, and vascular invasion all fall into the same sub-group, and the insular and anaplastic carcinoma 2 areas also sub-group together. However the classic PTC 2 area groups away from the other tumour foci that are close to it and aligns itself with the other PTC area. This may indicate that these two foci, which both contain the *BRAF *mutation, possess similar genetic profiles and may suggest that they are clonal in origin and have originated from the same mutational event. However, the anaplastic carcinomas separate into the two sub-groups within the cancer cluster perhaps indicating that these cancers may have arisen independently. Therefore one might speculate that it may not be a question as to whether multifocal tumours arise due to the clonal metastasis of tumour cells or of independently mutational events, but perhaps both phenomena can occur simultaneously within the one tumour to enhance cancer progression.

The ability of the miRNA profiles to distinguish the classic PTC foci from all other cancer areas, and the two more aggressive types of cancer from each other (Tables 2 &3) enforces the concept of divergent miRNA profiles within disparate foci of a multifocal tumour. Previous studies involving miRNA expression profiling have compared tumour to normal tissue to determine the differentially regulated miRNAs and in turn potential biomarkers [18]. Due to the many types of thyroid cancer, miRNA profiles have been examined in several different thyroid tumours [21]. However no study has investigated miRNA expression within a multifocal tumour. The varying miRNA profiles in the different foci of this tumour highlight a caveat that ought to be observed in the search for the discovery of future cancer biomarker signatures, particularly in the context of multifocal tumours.

This body of work also invokes a debate as to the practical uses of this emerging technology. Are these small RNAs to be considered solely as biomarkers as discussed above, or should we delve deeper into the functions of these regulators to enhance our understanding of disease processes? For instance, do the miRNAs respond to the cellular changes observed when a normal cell transforms into a cancer cell, or are they intrinsic mediators in this transformation. It has been previously suggested that miRNAs may possess oncogenic or tumour suppressor roles in the cell. For research in this field to progress, advancements must be made in the discovery of the gene targets of the miRNAs. For this reason we endeavoured to predict the gene targets of some of the miRNAs found to be differentially regulated in this study. These predicted results generated some biologically plausible pathways that may be influenced by miRNAs in the development of this cancer. Pathways such as angiogenesis and Wnt signalling are particularly relevant when considering the biology of this cancer due to their previous associations with this disease.

Components of the Wnt signalling pathway such as β-catenin and APC have been found to be mutated in many cancers including thyroid cancer [38]. The pathway can be hyperactivated due to DNA methylation or specific mutations resulting in the stimulation of cancer progression via the up-regulation of multiple genes involved in cell cycle progression, invasion, motility, and differentiation [39]. Aberrant activation of the Wnt pathway has been linked to the dedifferentiation of thyroid cells, a process universally observed with anaplastic thyroid cancer [40]. This pathway could have a role in the dysregulation of the cytoskeleton through the disruption of the cadherin-catenin complex comprised of e-cadherin, β-catenin, and α-catenin. Disruption or loss of the complex plays a prominent role in cancer and leads to loss of cell-cell adhesion, cytoskeletal remodelling, and increased migration in a process related to epithelial to mesencymal transition (EMT). EMT is a common occurrence in cancer progression and plays an important role in metastasis [41]. The majority of carcinomas (including thyroid cancer) lose e-cadherin mediated cell-cell adhesion. Smyth and colleagues [42] investigated e-cadherin mRNA expression in various thyroid tissue types and found a decrease in expression levels with decreasing differentiation. The up-regulation of the Wnt signalling pathway observed in this study and the reported loss of e-cadherin expression in anaplastic cancer could culminate in the disruption of the cadherin-catenin complex may be an important factor in the dedifferentiation and malignancy of this cancer.

Angiogenesis is also a prevalent feature in thyroid cancer, and one of the hallmarks of cancer. Mutation of the tumour suppressor p53 is commonly found in anaplastic cancer and was observed here (Figure 4). Hassan & colleagues [43] showed that secretion of VEGF was decreased by 34% in undifferentiated thyroid cancer cells in which mutated p53 has been knocked down; implicating this protein in angiogenesis. There have also been studies showing the ability of exogenous wild type p53 to increase chemosensitivity in ATC cell lines. It has been suggested that the combination of chemotherapy with wild type p53 gene therapy may be efficacious in treating ATC [37]. Therefore, although the results of gene targets analysis are speculative at this stage, they may provide insight into miRNAs' role in the mechanisms of cancer progression and provide inspiration for further research.

## Conclusion

The genetic analysis performed on the multifocal thyroid tumour areas consisting of divergent morphologies indicated that the insular and anaplastic tumours did not arise from PTC progenitor foci. However, due to the discordance in conclusions in other studies examining this issue, the miRNA profiles of the tumour areas were examined in an effort to shed further light on the tumourigenesis process. This analysis provided an interesting interpretation to the answer of the clonality question. Similarities were seen among the tumour areas (which must be anticipated due to the close proximity of the foci) but differences were also noted. The hierarchical clustering analysis indicated that the multiple foci may not have arisen due to the clonal metastasis of tumour cells or of independently mutational events, but perhaps both phenomena can occur simultaneously within the one tumour to enhance cancer progression.

This study illustrates the potential variability of miRNA expression in different tumour micro-environments. It highlights the importance of considering and reporting the areas of the tumour from which cells are excised for the study of miRNA expression. In light of this information, studies reporting miRNA signatures specific for disease types may need to be refined to allow for or exploit this variability. Studies may choose to report global expression patterns for whole sections of tumour, or alternatively focus on miRNA patterns that associate with propensity for metastasis – e.g. miRNAs altered in vascular areas, or perhaps those miRNAs with altered expression in hypoxic areas of tumour – which might correlate with resistance to chemotherapeutic intervention. The latter approach has particular promise in a translational medicine setting where a personalised approach to medicine may be indicated.

Another approach might be to investigate the differentially regulated miRNAs harvested from nominated areas (e.g. areas exhibiting lymphocytic infiltration or vascular invasion) of different types of tumours to determine whether they are universal markers of these features, or specific to lymphocytic infiltration or increased vascularisation in the thyroid.

Finally, when considering the best potential applications of miRNAs, either as biomarkers or biological mediators of disease, the answer remains to be seen. Further research is required to provide both solid information on the gene targets of these RNAs and robust miRNA biomarker signatures.

## Competing interests

No conflict of interest declared. Support was provided by Applied Biosystems for consumables used in this study; however, neither the researchers nor the company had any vested interest in the outcome of the project.

## Authors' contributions

SA sourced the archival tissue sample, performed laser capture microdissection, nucleic acid extraction, TaqMan expression analysis, and data analysis. PS participated in the data analysis and study design. RF reviewed and classified the archival tissue sample and the immunohistochemistry slides. SR performed the immunohistochemistry. KD, JL, and SG provided technical support. JOL & OS conceived the study and participated in its design and coordination.

## Supplementary Material

Additional file 1 Supplemental data. miRNA RQ values. Click here for file
